# Phytoremediation of Soils Contaminated with Heavy Metals from Gold Mining Activities Using *Clidemia sericea* D. Don

**DOI:** 10.3390/plants11050597

**Published:** 2022-02-23

**Authors:** Elvia Valeria Durante-Yánez, María Alejandra Martínez-Macea, Germán Enamorado-Montes, Enrique Combatt Caballero, José Marrugo-Negrete

**Affiliations:** 1 Water, Applied, and Environmental Chemistry Research Group, Department of Chemistry, Faculty of Basic Sciences, University of Córdoba, Montería 230002, Colombia; evdurante@correo.unicordoba.edu.co (E.V.D.-Y.); mariamartinezm@correo.unicordoba.edu.co (M.A.M.-M.); genamoradomontes@correo.unicordoba.edu.co (G.E.-M.); 2 Department of Agricultural Engineering and Rural Development, Faculty of Agricultural Sciences, University of Córdoba, Montería 230002, Colombia; emcombatt@correo.unicordoba.edu.co

**Keywords:** cadmium, lead, native species, mercury, phytostabilization

## Abstract

Soils contaminated by potentially toxic elements (PTEs) as a result of anthropogenic activities such as mining are a problem due to the adverse effects on human and environmental health, making it necessary to seek sustainable strategies to remediate contaminated areas. The objective of this study was to evaluate the species *Clidemia sericea* D. Don for the phytoremediation of soils contaminated with PTEs (Hg, Pb, and Cd) from gold mining activities. The study was conducted for three months, with soils from a gold mining area in northern Colombia, and seeds of *C. sericea*, under a completely randomized experimental design with one factor (concentration of PTEs in soil) and four levels (control (T0), low (T1), medium (T2), and high (T3)), each treatment in triplicate, for a total of twelve experimental units. Phytotoxic effects on plants, bioconcentration (BCF), and translocation (TF) factors were determined. The results obtained for the tissues differed in order of metal accumulation, with the root showing the highest concentration of metals. The highest values of bioconcentration (BCF > 1) were presented for Hg at T3 and Cd in the four treatments; and of translocation (TF > 1) for Hg and Pb at T0 and T1; however, for Pb, the TF indicates that it is transferable, but it is not considered for phytoextraction. Thus, *C. sericea* demonstrated its potential as a phytostabilizer of Hg and Cd in mining soils, strengthening as a wild species with results of resistance to the stress of the PTEs evaluated, presenting similar behavior and little phytotoxic affectation on the growth and development of each of the plants in the different treatments.

## 1. Introduction

Soil contamination with potentially toxic elements (PTEs) from anthropogenic activities such as mining has become a global concern because of the impacts on human and environmental health due to toxicity, persistence, and bioaccumulation in the trophic chain [1]. Different countries face PTEs soil contamination, but differ in the knowledge of the problem, treatments, and technologies to solve them [2]. Reports indicate that worldwide, there are more than five million sites with soil contamination by PTEs [3]. In Colombia, in the last decade, different investigations have shown high concentrations of PTEs in soils of gold mining areas [4,5,6,7,8,9,10,11].

The most common PTEs in contaminated soils from gold mining processes are Hg, Pb, and Cd [12,13]. Accumulation in soils causes alterations in their physical, chemical, and biological properties, causing deterioration of the ecosystem services provided by this non-renewable natural resource [14,15,16]. Likewise, agricultural activities in these contaminated soils are of great concern because metals can translocate to the edible parts of plants and impact crop growth and development due to phytotoxicity, becoming a risk to food safety due to the toxic effects generated by these elements on human health [13]. Hence, the importance of seeking environmental solutions to remediate and/or restore contaminated soils.

Different physical, chemical, and biological treatments exist to remove PTEs from soils [17]. At present, phytoremediation has been shown to be an effective, environmentally friendly, non-invasive, aesthetically pleasing, and economically viable technique to remediate soils contaminated with PTEs [18,19]. This technology is based on using plants capable of tolerating, accumulating, removing, or immobilizing pollutants such as PTEs [17]. Phytoremediation uses different mechanisms that differ from the physiological processes of plants, phytoextraction and phytostabilization being the most used for soil remediation [20]. These techniques have been extensively evaluated at the greenhouse scale [4,21,22,23], tested on in situ pilots [11,24,25,26,27], and in field trials [28,29,30,31,32]. The use of this technology has shown its ability to treat contamination and stop the disturbance, and restore or recover the structural and functional integrity of affected ecosystems [33,34]. The selection of plant species is an important factor in applying phytoremediation techniques because plants must have certain characteristics that allow them to grow in specific site conditions [35,36]. In many cases, they have used alien plant species that can become invasive and deteriorate the native flora, representing a threat to ecosystems [1,37]. Different studies propose the use of native plants growing in areas to be remediated and/or restored for the environmental benefits and reduction of treatment costs [35,38,39]. It is important to highlight that plants growing in contaminated areas have higher resistance through more efficient exclusion or higher accumulation and tolerance to potentially harmful concentrations of contaminants than those plants growing in uncontaminated soils [37,40].

*Clidemia sericea* D. Don is a plant of the Melastomataceae family, usually found in disturbed sites, and can grow up to 2000 masl [41]. This species has not been evaluated in experimental phytoremediation trial. However, Marrugo-Negrete et al. [5], at an inventory of native plants from a mining site, reported for the genus *Clidemia* sp. a translocation factor of 1.43 for Hg, indicating that this metal can be transferred from the root to the plant shoots. Chamba et al. [42], for the species *Miconia zamorensis* Gleason belonging to the Melastomataceae family as *C. sericea*, evidenced high concentrations of Pb and Cd in root, stem, and leaves. This indicates that this species may have the potential for phytoremediation of PTEs (Hg, Pb, and Cd); in this sense, the evaluation of this species under controlled conditions in a pot assay leads to the generation of valuable information over the quantity of toxic elements that accumulated in the plant organs to restore polluted sites. The objective of the present study was to evaluate the phytoremediation process using a native plant, *C. sericea*, growing in soils contaminated with three levels of PTEs (Hg, Pb, and Cd) near gold mining activities in northern Colombia. The phytoremediation performance was evaluated by measuring the concentration of Hg, Pb, and Cd in different parts of the plant, such as root, stem, and leaves. Two phytoremediation indices were calculated, the Translocation Factor (TF) and the Bioconcentration Factor (BCF), while plant tolerance and development were evaluated by following the length, diameter, number of leaves, dry biomass, and pigments.

## 2. Results and Discussion

### 2.1. Physicochemical Characteristics of Soils

The physicochemical characteristics of the soils in their initial and final conditions are presented in Table 1. The treatments represent the four levels of PTE concentration. T0 (control treatment) corresponds to a soil far from mining activities and with little anthropic intervention. T1, T2, and T3 correspond to soils with low, medium, and high concentrations of PTEs from a mining area, respectively. The four soils presented a clay loam texture and low organic matter (OM). Treatments T2 and T3, corresponding to medium and high metal concentrations, can be classified as acid sulfate soils due to their extremely acidic condition (pH: 3.5–4.5) and high sulfur contents (>20 mg kg^−1^) [43]. Treatments T0 and T1 presented a pH higher than 5, high phosphorus concentration (>80 mg kg^−1^), and relatively low sulfur and exchangeable aluminum values compared to T2 and T3. 

Some chemical parameters showed significant variations between initial and final values (*p* < 0.05). Except for T2, at the end of the experiment, there was an increase in soil pH. Salas-Moreno and Marrugo-Negrete [22] reported an increase in pH in the course of phytoremediation of Pb and Cd contaminated mining soils using *Paspalum fasciculatum* Willd. ex Flüggé; Kim et al. [44] reported the increase of pH in acid soils contaminated with Cd, Pb, Zn, and Cu after phytoremediation with *Brassica juncea* (L.) Czern. The increase in soil pH is likely related to nitrogen uptake predominantly in the form of NO_3_-N, with simultaneous excretion of OH^-^ ions to maintain electrical neutrality within plant roots [45,46]. The release of H^+^ or OH^−^ ions by roots can modify rhizosphere pH and increase or decrease metal dissolution because H^+^ ions can displace PTE cations adsorbed on soil particles [22,46]. 

The OM at the end of the experiment for all treatments increased compared to the initial conditions, being significantly higher for T0, T1, and T3 (*p* < 0.05). This is explained by the exudates that are released by the roots in the rhizosphere during plant growth, which are composed of sugars, organic acids, amino acids, fatty acids, flavonoids, secondary metabolites, especially phenolics, polysaccharides, proteins, among others, most of which have carbon and nitrogen groups that are part of the composition of the OM. Likewise, these compounds are synthesized, accumulated, and secreted in the process of respiration, radical hair shedding, and root elongation [47,48]. 

At the end of the experiment, sulfur (S) increased significantly compared to the initial value (*p* < 0.05). The treatments that presented the highest increase were the soils closest to the area of the mining process (T2 and T3). Likewise, phosphorus (P) content increased significantly in T0 and T3 (*p* < 0.05), and in T1, it decreased significantly compared to the initial value (*p* < 0.05) and without significant changes in T2. Variations in S and P are due to soil chemical dynamics, plant influence, and environmental conditions. In mining soils decomposing primary materials and elements in changing phase, such as sulfides and phosphates, tend to solubilize when interacting with plant root exudates and plant irrigation water [49,50]. Plants can add S to the soil by exudates in the rhizosphere [46,51], which explains the higher concentrations at the end of the experiment. 

The cation exchange capacity (CEC), given by the sum of Ca^2+^, Mg^2+^, K^+^, Al^3+^ + H^+^, cations, increased in the treatments after the phytoremediation process, being significant in T2 and T3 (*p* < 0.05). The irrigation water, nutrient and water uptake by roots, ion release by roots, and rhizosphere microorganisms may explain the changes in soil chemical properties after the phytoremediation process [46,52]. 

### 2.2. Concentration and Bioavailability of PTEs in Soil

For each treatment, the concentrations of Hg, Pb, and Cd in soil decreased significantly after the phytoremediation process compared to their initial condition (*p* < 0.05, Table 2). The initial concentrations and after the phytoremediation process presented the following order Pb > Cd > Hg. The concentrations of PTEs were much higher than values reported for non-contaminated soils in Colombia (Hg: 0.028 ± 0.07 mg kg^−1^, Pb: 0.012 ± 0.01 mg kg^−1^ and Cd: 0.008 ± 0.001 mg kg^−1^ [53]). In addition, in the different treatments, a decrease in the concentration of PTEs was observed after the process, as in other studies in different species, such as *Jatropha curcas* L., *B. juncea* for Hg [11,54]; *Sida acuta* Burm. f. for Pb [55]; *Thlaspi caerulescens* J.Presl & C.Presl, *Arabidopsis halleri* (L.) O’Kane & Al-Shehbaz, *Nicotiana tabacum* L., for Cd [56,57,58].

Regarding bioavailability, Cd in all treatments presented the highest percentage compared to Hg and Pb, being significantly higher after the process (*p* < 0.05; Table 2). Pb presented the lowest bioavailability (<1%) in all treatments. After the phytoremediation process, except for Hg at T0, the bioavailable percentage for Hg and Pb was less than 1%. For Hg, it decreased at T0, T1, and T3 (*p* < 0.05); for Pb, it remained stable at T0 and T1, decreasing significantly in the other two treatments (*p* < 0.05). The results show that a higher total concentration of a metal does not imply a higher bioavailability (Table 2) because it depends on the soil characteristics, the mineralogical composition, and the environmental conditions of the soil, among other factors [7,59,60,61].

The high bioavailability of Cd is possibly due to it being highly soluble under acidic conditions as free water ions (bound only to water molecules) [62]. In addition, soil microbial activity and plant root exudates through the release of organic acids cause the solubilization of minerals containing this metal, which favors its increase, as presented in each treatment [48,63]. Regarding Pb, its low availability is because most of the compounds of this metal have low solubility and precipitate very easily by sulfates and phosphates, which makes it one of the least soluble PTEs in soil [62,64]. Hg increases its bioavailability at acidic pH because free H^+^ ions combine with colloidal substances that have a negative charge and Hg^2+^ will be released into the liquid phase of the soil [65]. However, in the presence of sulfur, it can decrease its bioavailability by forming sulfur compounds that are stable and insoluble to be accumulated or translocated to plants [66]. Likewise, other studies have evidenced the low bioavailability of Hg and Pb in agricultural soils, mining soils, or soils affected by mining activities [53,67,68,69,70,71,72,73,74]. However, Li et al. [75] showed higher results than this study, 3.63% and 15.4% for Hg and Pb, respectively, after phytoremediation with *Solanum nigrum* L. for Cd, bioavailabilities of 53.2–69.1% [65] and 9.85% [76] have been reported for mining soils in China. Bioavailability percentages of 46.3% have also been reported in mining soils with spontaneous vegetation [77] and around 25% in phytoremediation of mining soils with *Paspalum fasciculatum* Willd. ex Flüggé [22], values similar to those of this study.

### 2.3. Growth Behavior of C. sericea

**Figure 1:**
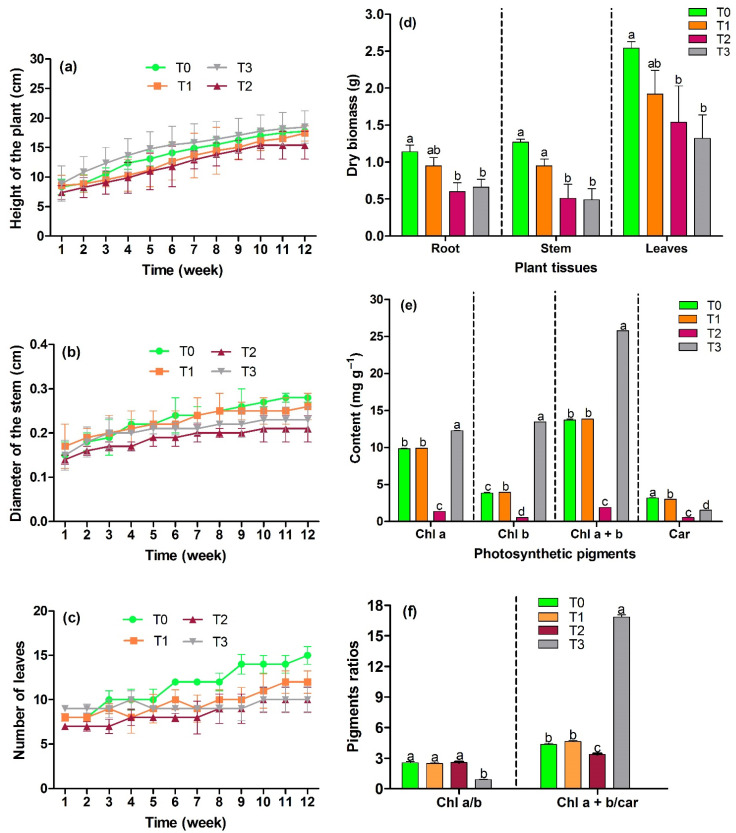
Behavior of morphometric parameters, growth, and dry biomass of *C. sericea*. (**a**) Height of the plant. (**b**) Diameter of the stem. (**c**) Number of leaves. (**d**) Dry biomass per tissue for each treatment, total biomass is the sum of tissues per treatment. (**e**) Photosynthetic pigments. Chlorophyll a. Chlorophyll b. Chlorophyll a + b. Carotenoids. (**f**) Pigments ratios. Chlorophyll a/b. Chlorophyll a + b/carotenoids. Different letters indicate significant statistical differences (*p* < 0.05) using Tukey’s test between each treatment per variable.

The growth behavior of *C. sericea* during the phytoremediation process was ascending for plant height, stem diameter, and the number of leaves (Figure 1a–c; Figure S1). The development of these variables in T1, T2, and T3 was relatively low compared to T0, except for plant height, which was higher in T3. In this study, phytotoxicity was shown by necrosis and leaf drop for T2 and T3 (Figure S2), at 24 days after planting, so it could be a response of the plants to Hg, Pb, and Cd exposure as part of their process of tolerance and adaptation to the environment during the vegetative stage of some plants [78,79,80,81].

The root, stem, leaf, and total dry biomass are shown in Figure 1d (Table S1). Total biomass presented reductions of T1: 22.62%, T2: 46.67%, and T3: 50.10%, compared to the control (T0). The reduction in biomass, plant height, stem diameter, and the number of leaves is explained by the toxic soil conditions since plants require additional energy to counteract PTEs stress [4,82]. Additionally, the inhibition of root development and the imbalance of water and nutrient transport leads to growth inhibition, damage to the structure, decrease of physiological and biochemical activities that affect biomass production [83,84,85]. In addition, soils presenting acid sulfate characteristics limit the availability of nutrients and inhibit the absorption of exchangeable bases for plants affecting their growth, development, and productivity [86,87,88]. Other studies have shown the reduction of biomass, plant height, stem diameter, and the number of leaves in different species due to stress caused by PTEs [4,83,89,90,91,92]. However, Salas and Marrugo [22] and Dinu et al. [93], in their studies of Cd and Pb exposure of *P. fasciculatum* and *Ocimum basilicum* L. species, respectively, induced a greater plant height in soils with higher concentration compared to the control, and even the decrease in stem diameter and the number of leaves was minimal, indicating the stimulation of plant growth at higher concentration of PTEs; explaining the results obtained for T3. 

The photosynthetic pigments chlorophyll a (Chl a), b (Chl b), a + b (Chl a + b), a/b (Chl a/b), carotenoids (Car), chlorophyll a + b/carotenoids (Chl a + b/car) (Figure 1e,f, Table S1), presented significant differences among treatments (*p* < 0.05), being higher in T3 and lower in T2, and for carotenoids, higher in T0 and T1. The lower content of these pigments in T2 can be explained by a higher incidence of PTEs in reducing the number of chloroplasts or a structural disruption of the chloroplast, accelerating chlorophyll degradation and affecting photosynthetic capacity [94,95,96]. In this treatment, Cd presented higher bioavailability, which inhibits the enzymes responsible for chlorophyll biosynthesis, i.e., 5-aminolevulinic acid dehydration and protochlorophyllide reductase or its degradation due to the formation of free radicals of polyunsaturated fatty acids as a result of increased lipoxygenase activity [97]. This PTE hinders the division of chloroplasts, their growth and arrangement of the thylakoid system, affecting carotenoid content [98]. Cui et al. [99] reported a negative effect of soil-available Cd on chlorophyll (a + b) and carotenoid activities in *Amaranthus Hypochondriacus* L. leaves. Pb induces an alteration of chloroplast ultrastructure and displaces Ca^2+^ and Mn^2+^ from the light-harvesting complex of photosystem II; this photosystem is also affected by Hg as it replaces the central chlorophyll atom, magnesium, which prevents photosynthetic light-harvesting by altering the photosynthesis process [98,100]. The adverse effect on photosystem II in the thylakoids causes a decrease in carotenoid contents [101]. On the contrary, in T3, a higher chlorophyll content was presented, managing to maintain normal photosynthesis under the stress caused by exposure to PTEs, which is directly reflected in plant growth [96,97,102]. In some reports, the photosynthetic pigments evaluated showed an increase after exposure to PTEs, as was the case for T3 [103,104,105,106]. T0 and T1 presented similar chlorophyll and carotenoid contents, indicating that the lower PTEs concentration did not negatively affect the pigments of T1 plants. The contradictory results shown for T3 compared to T0 can be explained by the effects of the combination and interaction of PTEs in the treatments because they are competing with each other in the soil medium [106]. The Chl a/b ratio was similar in T1 and T2 compared to T0 (control), presenting values close to 3, except T3 with a value < 1. The results indicate for T1 and T2 a decrease in chlorophyll due to the stress generated by PTEs, and for T3 a final decomposition of chlorophyll, due to the toxicity of the elements that can reduce the size of the peripheral part of the antenna complex. The Chl (a + b)/car ratio presented similar values for T0 and T1, being lower for T2 and a high value for T3, showing for T2 a strong reduction of chlorophyll compared to carotenoids, and for T3 a pronounced reduction of carotenoids compared to chlorophyll [107]. Different authors have reported on the content of photosynthetic pigments, which vary according to the species, the concentration of PTEs, and the degree of toxicity of these individually and mixed. Chinmayee et al. [108] reported variation in chlorophyll a, b, a/b, and carotenoid contents in response to different PTEs to which *Amaranthus spinosus* L. was exposed, with Pb increasing pigment contents. Leal-Alvarado et al. [109] reported no differences in the contents of chlorophyll a, b, and carotenoids in *Salvinia minima* Baker evaluated at different Pb concentrations. Zhan et al. [110], for *Nicotiana tabacum* L. leaves exposed to Cd reported Chl a/b values close to 3 in their treatments similar to the control. Fargašová and Molnárová [107], for *Sinapis alba* L. under PTEs stress, obtained in all cases a similar Chl a/b ratio and Chl (a + b)/car values lower than the control. 

### 2.4. Concentration of Hg, Pb, and Cd in Plant Tissues and Phytoremediation Indices

**Figure 2:**
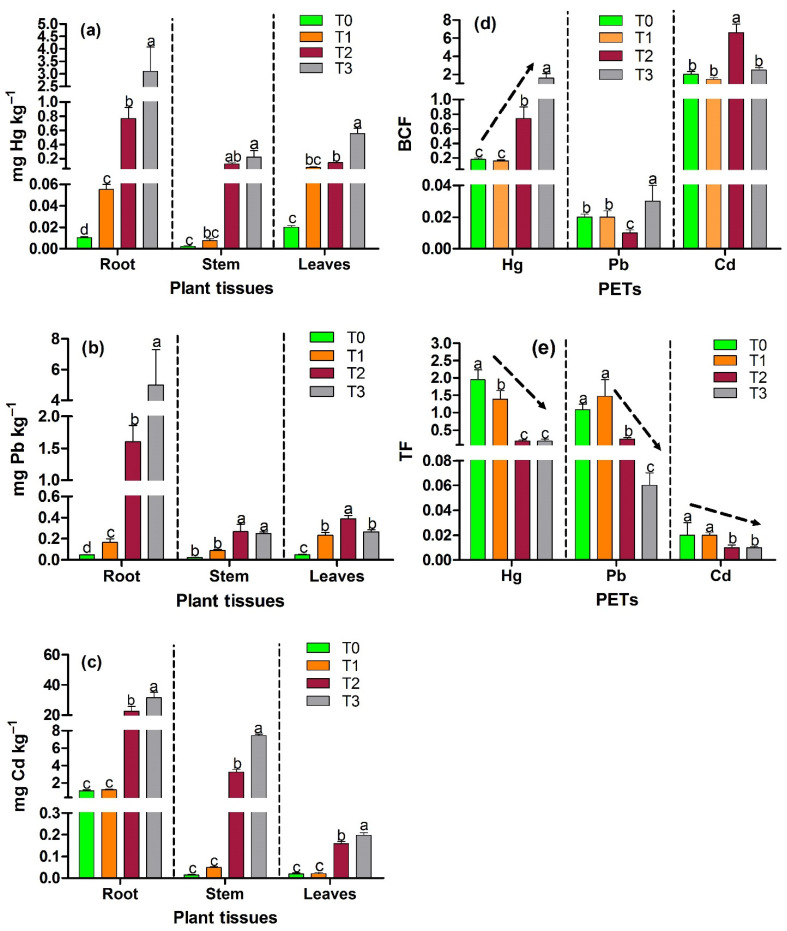
Concentration of PTEs in plant tissue and phytoremediation indices (**a**) Hg. (**b**) Pb. (**c**) Cd. (**d**) BCF. (**e**) TF. Different letters indicate significant statistical differences (*p* < 0.05) using Tukey’s test between each treatment per tissue.

The concentrations of Hg, Pb, and Cd in root, stem, and leaves showed significant differences among treatments (*p* < 0.05, Figure 2a–c). The accumulation of PTEs in plant tissues presented the following order for Hg and Pb: T0, T2, and T3: Root > Leaves > Stem; T1: Leaves > Root > Stem. For Cd, it presented the order for T0: Root > Leaves > Stem; T1, T2, and T3: Root > Stem > Leaves. Plant tissues showed a higher accumulation of PTEs following the order of highest to lowest concentration in the treatments: T3 > T2 > T1 > T1 > T0. In general, the highest concentrations of metals were present in the root compared to stem and leaves, with maximum values at T3 for Hg, Pb, and Cd of 3.11 ± 0.97 mg kg^−1^, 4.99 ± 2.32 mg kg^−1^, and 31. 57 ± 3.37 mg kg^−1^, respectively; in leaves and stem the concentrations were <1 mg kg^−1^, except for Cd concentration in stem for T2 (3.25 ± 0.32 mg kg^−1^) and T3 (7.44 ± 0.16 mg kg^−1^).

Plants tend to accumulate Hg, Pb, and Cd in the roots followed by the shoots [5,10,22,82,111,112,113,114,115,116]. This tendency of roots to accumulate most of the bioavailable metals is due to the fact that these in the process of absorption of water and essential nutrients are in direct contact with the Hg, Pb, and Cd present in the soil and accumulate in the cell walls of the root by the attraction between their negative charge and the positive charge of metals [116,117]. This prevents toxic effects (necrosis and chlorosis) in the aerial parts of the plant [82,115]. Additionally, Cd accumulation in roots may also likely be due to the combination with sulfur-rich peptides or other constituents (such as organic acids) from the cell sap in the root vacuole, considered to be predominantly a sink for Cd [118,119]. Leaves showed a higher accumulation of Hg and Pb than stems, which could be due to the function of the latter to transport liquids and nutrients between roots and leaves through xylem and phloem, which does not allow Hg and Pb to accumulate in this tissue, facilitating their storage in leaves, which are the final receptors [5,116]. In addition, vacuoles in leaves are large Pb-accumulating organelles [120]. The higher Hg accumulation that occurred in leaves at T1 (low concentration) was similar to what was found by Concas et al. [121] and Marrugo-Negrete et al. [5] because volatilized Hg can be captured by leaf stomata [5]. 

On the other hand, for species of the Melastomataceae family to which *C. sericea* belongs, accumulation of Hg, Pb, and Cd has been reported. Marrugo-Negrete et al. [5], presented for *Clidemia* sp. Hg concentrations in their tissues (Root: 0.23 mg kg^−1^, Stem: 0.11 mg kg^−1^, Leaves: 0.22 mg kg^−1^) results similar to those obtained in T2 and T3; and Chamba et al. [42], for *M. zamorensis* showed high Pb concentrations in root: 379 mg kg^−1^, stem: 67 mg kg^−1^ and leaves: 68 mg kg^−1^, higher than our study, and Cd concentrations in root (2.8 mg kg^−1^), stem (0.89 mg kg^−1^) and leaves (0.80 mg kg^−1^), similar to those obtained for leaves and stem in our study.

The BCF and TF values (Figure 2d,e) were calculated to determine the ability of plants to accumulate metals in their roots from the soil and translocate them to aerial tissues (leaves), respectively. BCF > 1 were obtained for Hg in T3 and for Cd in the four treatments. TF > 1 were obtained for Hg and Pb at T0 and T1, this can be explained by the low concentrations of these elements in the soil that are usually a limiting factor for the accumulation of substantial amounts in the roots, which allows the translocation of these metals through the development of plant detoxification mechanisms based on the sequestration of ions in the vacuole by binding with ligands (proteins, organic acids, and peptides), resulting in high translocation values [122,123]. The highest BCF values were presented for Cd, which is due to the fact that this metal is more mobile with respect to Hg and Pb, and therefore bioconcentrates more easily in plant roots [90]. The TFs for T2 and T3 approaching 0.1 indicate the exclusion of the element in plant tissue at higher concentrations of the PTEs evaluated [124]. This can be explained as a plant mechanism that consists of altering the permeability of membranes, changing the capacity of cell walls, or exuding more chelating substances to maintain the physiological concentrations of essential metal ions and to minimize exposure to non-essential heavy metals; avoiding toxic effects on aerial tissues [79]. Marrugo-Negrete et al. [4] reported similar behavior in *J. curcas* for TFs in higher Hg concentration treatments. Most plant species present a restriction of translocating Pb and Cd from roots to shoots, so in many cases, the TF is lower than 0.07, as presented in the results [98]. 

Thus, taking into account the criteria of phytoextraction and phytostabilization potential according to the values of BCF and TF [4,108]; it can be determined that the species *C. sericea* has phytostabilizing potential for Hg and Cd in soils. In the case of Pb, it can be evidenced that it is highly transferable at T0 and T1 (TF > 1); however, the species is not phytoextractive of this metal because it does not meet the aforementioned criterion by presenting BCF < 1 (<0.03). Other studies have reported BCF and TF for the PTEs evaluated in native species growing in mining areas with potential use for phytostabilization, phytoextraction or metal tolerance [125,126]. Marrugo-Negrete et al. [5] reported similar BCF (0.36) and TF (1.43) values for Hg for *Clidemia* sp. at T0 and T1. Chamba et al. [42] reported for *M. zamorensis* BCF (Pb: 0.24–0.59 and Cd: <0.06–1.01) and TF (Pb: 0.17–0.19 and Cd: <0.05–0.29) values, similar TF > 0.1 for Cd in the four treatments, similar TF for Pb at T2, and similar BCF (1.01) to T1 for Cd. Demonstrating that the accumulation and translocation capacity of these metals are presented in different proportions, depending on the concentration and bioavailability of metals, physicochemical characteristics of the soil such as pH and OM content, in addition to the cellular mechanisms of plants to avoid or tolerate the absorption of metals [113,127,128]. 

### 2.5. Correlation between Soils Parameters and Phytoremediation Indices

Statistically significant correlations (*p* < 0.05) were found between Hg, Pb, and Cd concentrations in soil and different plant tissues (Table 3), showing that Hg, Pb, and Cd accumulated in tissues is directly related to the content of metals in the soil. The highest correlations were found for Hg in leaves and root, and for Pb and Cd in the stem; presenting the following order, Hg: Leaves > Root > Stem, Pb and Cd: Stem > Root > Leaves; showing a similar pattern for Pb and Cd, differing from that obtained for Hg. These results could suggest that *C. sericea* does not use any mechanism that excludes or restricts the uptake of metals by the roots for the different concentrations of Hg, Pb, and Cd. The relationship between the concentration of PTEs accumulated by the tissues of a plant species and the concentration in the soil may depend on the taxon [129], presenting positive or negative correlations, significant or not, depending on the interaction of the species. Marrugo-Negrete et al. [5,82], Galal et al. [130], do Nascimento Júnior et al. [131], and Eid et al. [132] have shown significant positive correlations between the accumulation of PTEs in tissues of different species with the concentrations of these in the soil, showing that the metal concentrations in plants frequently increase as their concentration in the soil increases.

Both Hg phytoremediation indices estimated showed significant relationships with pH, OM, S, P, and CEC; the same results were found only for the TF index of Pb and Cd with all these soil parameters. In the case of Pb, which is the PTE with the lowest bioavailability of this study, the BCF estimated through PTE concentration in soil, no significant correlations were found. Soil sulfur was highly inversely correlated with the TF of the three elements, while OM, pH, phosphorus, and CEC were positively correlated with this index. Sulfur is used as a soil amended for heavy metals phytoremediation because it decreases pH and solubilizes the metals, promoting phytoextraction [133]. However, we observed an opposite result because the accumulation of metals to aboveground tissue decreases with the Sulfur content in the soil.

## 3. Materials and Methods

### 3.1. Soil Sampling

Soils were collected in a gold mining process area located in northern Colombia with coordinates 8°57′6.03″ N, 74°6′24.94″ W; 8°57′3.73″ N, 74°6′23.68″ W; and 8°56′57.14″ N, 74°6′11.70″ W. Samples were taken at a depth of less than 30 cm [134]. The samples were taken in a transect in a longitudinal direction to the mine waste area: 1 km (T1), 300 m (T2), and 20 m (T3), respectively; for each treatment, 150 kg of soil were collected. The control treatment (T0) 150 kg of soil were taken from a reference site far from the mining process and with little intervention by anthropogenic activities (coordinates 8°47′36.80″ N and 75°51′42.56″ W). Next, the soils were transported to the laboratory, air-dried, homogenized, and passed through a 2.0 mm sieve [135]; subsequently, samples were taken for physicochemical analysis and arranged in the experimental units.

### 3.2. Seedling Production

A seedbed of the species *C. sericea* was made using seeds collected in a rural area close to where mining activities take place. These seeds were germinated in soil free of Hg, Pb, and Cd in germination trays. Four weeks after germination, seedlings with the highest vigor, number of leaves, and average stem length (>10 cm) were selected for transplanting into the experimental units. The taxonomic identification of this species was carried out in the Herbarium of the University of Córdoba, and the plant material was deposited under the code HUC 8205.

### 3.3. Experimental Design and Greenhouse Trial

A completely randomized experimental design was used with one factor (PTEs concentration in soil) and four levels (control, low, medium, and high concentration). Each treatment was carried out in triplicate, for a total of twelve experimental units. The treatments were designated as T0, T1, T2, and T3, corresponding to control, low, medium, and high concentration of Hg, Cd, and Pb, respectively. The response variables were Hg, Pb, and Cd concentrations in roots and shoots, growth performance (dry biomass), and photosynthetic pigments (chlorophyll and carotenoid content).

The phytoremediation process was carried out in a greenhouse, under environmental conditions with an average temperature of 28.9 ± 3.04 °C and relative humidity of 69 ± 8.83%, where 45 kg of homogenized soil were placed in waterproofed wooden structures measuring 50 × 50 × 30 cm, with a volume of 75 L and an area of 0.25 m^2^ for each unit. Then, four plants were transplanted randomly in these units after having passed the seedling stage. All plants were irrigated with three-quarters of the field capacity to avoid leaching [4]. Experimental units (Figure S3) were randomly arranged such that all had uniform illumination [136].

The experiment lasted 96 days, and every eight (8) days phytotoxic effects were monitored, observing whether wilting, chlorosis, necrosis, and leaf drop occurred. In the end, the plants were harvested and washed with deionized water to eliminate soil particles that adhered to the surface.

### 3.4. Dry Biomass and Pigments Determination

The plant tissues were divided into root, stem, and leaves. The dry weight of each tissue was taken, after being dried in an oven with an integrated timer for 4 days at 40 °C [5]. Subsequently, each tissue was grounded and homogenized before the analysis, using a Wiley-type mill (TECNAL reference TE-650/1). Chlorophyll and carotenoids were determined using the Lichtenthaler method [137], leaves were macerated with liquid nitrogen and placed in 80% acetone overnight; the supernatant was measured by UV-Vis spectroscopy with a Perkin Elmer Lambda 11 spectrometer at 663, 647, and 470 nm. The equations for the determinations of chlorophyll a (Equation (1)), chlorophyll b (Equation (2)), total chlorophyll (Equation (3)), and total carotenoid (Equation (4)) concentrations in leaf pigment extracts for (*v*/*v*) are as follows:

(1) $$\mathrm{Chl}\;a=12.25A_{663}-2.79A_{647}$$

(2) $$\mathrm{Chl}\;b=21.50A_{647}-5.10A_{663}$$

(3) $$\mathrm{Chl}\;a+b=7.15A_{663}+18.71A_{647}$$

(4) $$\mathrm{Car}=\frac{1000A_{470}-1.82\mathrm{Chl}\;a-85.02\mathrm{Chl}\;b}{198}$$

### 3.5. Soil Physicochemical Analysis

At the beginning and end of the experiment, dry and sieved (2 mm) soil samples were taken to evaluate soil physicochemical parameters. Soil texture was determined by measuring the proportions of clay (<0.002 mm), silt (0.002–0.05 mm), and sand (0.05–2 mm) particles present in the soil using the pipette method, and soil type was classified using the soil texture triangle [138]. Soil pH was determined at a 1:1 soil-distilled water ratio using a pH meter (WTW 330i) [53]. Organic matter (OM) content was determined by the Walkley–Black method [139,140]. Available sulfur (S) and phosphorus (P) contents were determined by the 0.008 M calcium monophosphate method [141] and the Bray II method [142], respectively, and cation exchange capacity (CEC) was calculated as the sum of Ca^2+^, Mg^2+^, K^+^, Na^+^, and Al^3+^ + H^+^, concentrations, determined by the 1.0 M ammonium acetate method at pH 7.0 [143].

### 3.6. Hg, Pb, and Cd Analysis in Soils and Plants

PTEs concentrations in soil and plant tissue samples were determined for Hg with a direct mercury analyzer (DMA-80 TRICELL; Milestone Inc., Sorisole (BG), Italy), using 0.3 g of sample, following EPA method 7473 [144]. For Pb and Cd, 0.3 g of sample were subjected to acid digestion in a microwave oven (Ethos One; Milestone) using an 8:2 mixture of HNO_3_:H_2_O_2_, by EPA method 3051A [145]. Concentrations were quantified by atomic absorption spectroscopy with a GFS35Z-Zeeman graphite furnace using a Thermo Scientific iCE 3500AA System.

For the bioavailability of Hg, Cd, and Pb in soils, the first phase extraction of the method developed by the European Community Reference Bureau (BCR) [146] was performed. First, 1 g of soil sample was taken, to which 40 mL of 0.11 mol L^−1^ acetic acid was added and shaken for 16 h at 25 °C on a mechanical shaker. The samples were centrifuged at 4000 rpm for 10 min, and the supernatant passed through a 0.45 mm filter. Subsequently, Hg quantification was performed by cold vapor atomic absorption spectroscopy (CVAAS) using a Thermo Elemental Solaar S4; for Cd and Pb, they were determined as the total concentration [53].

The analytical quality control of the methods used was evaluated in triplicate using certified reference materials for soil: CRM-008-050 from Resource Technology Corporation (RTC) and for plant: Trace and Minor Elements in Lichen IAEA-336 from the International Atomic Energy Agency (IAEA). The certified values and recovery percentages obtained for Hg, Pb, and Cd in soils and plants are shown in the supplementary information (Table S2). For all sample analyses, the coefficients of variation were less than 5%, and the values of the reference materials were within the 95% confidence limits. Ten percent of the samples were analyzed in duplicate. The limits of detection (LOD) obtained for soils: 0.1 µg Hg kg^−1^, 25 µg Pb kg^−1^, and 5 µg Cd kg^−1^; and for plants: 0.1 µg Hg kg^−1^, 80 µg Pb kg^−1^, and 8 µg Cd kg^−1^; calculated as three times the standard deviation of 10 measurements of a blank [147].

### 3.7. Bioconcentration and Translocation Factors in Plants

Bioconcentration (BCF) and translocation factors (TF) were determined for the different PTEs. The BCF Equation (5) was determined as the ratio of the metal concentration in the root to the total concentration in the soil [148]. The TF Equation (6) was calculated as the ratio of the concentration in the aerial organs (leaves) to the concentration in the root [149]. Considering BCF and TF, if BCF and TF > 1 the plant has the potential to be used in phytoextraction [4], and if BCF > 1 and TF < 1, the plant has potential for phytostabilization [108].

(5) $$\mathrm{BCF}=\frac{\left[\mathrm{Metals}\;\mathrm{in}\;\mathrm{the}\;\mathrm{root}\right]}{\left[\mathrm{Metals}\;\mathrm{in}\;\mathrm{the}\;\mathrm{soil}\right]}$$

(6) $$\mathrm{TF}=\frac{\left[\mathrm{Metals}\;\mathrm{in}\;\mathrm{the}\;\mathrm{leaves}\right]}{\left[\mathrm{Metals}\;\mathrm{in}\;\mathrm{the}\;\mathrm{roots}\right]}$$

### 3.8. Statistical Analysis

The results were expressed as the mean ± standard deviation of the triplicate determinations. The assumptions of normality (Shapiro–Wilk test) and homogeneity of variance (Levene’s test) were checked. Simple ANOVA, multiple comparisons of means with Tukey’s test, and Pearson’s correlations were performed to establish the relationship between the concentration of PTEs in soil and those in plant tissues and the relationship between chemical soil characteristics with BCF and TF phytoremediation indices, establishing a significant level of 95% (*p*-value < 0.05). The analyses were performed with Minitab Version 19, Advanced Analytics Software (SAS) Version 9.4, and the graphical outputs were performed with GraphPad Prism Version 5.

## 4. Conclusions

*C. sericea* showed a high tolerance to Hg, Cd, and Pb exposure in soils. The root was the tissue with the highest accumulation. The BCF and TF values indicate that this plant can be used for phytostabilization of Hg and Cd, and for Pb they indicate that it is transferable, but not considered for phytoextraction. Considering that *C. sericea* is a wild species that grows in mining areas, it showed resistance to stress generated by PTEs, presenting similar growth and development in the three treatments (T1, T2, and T3) compared to the control (T0). Likewise, there were few phytotoxic effects reflected in the morphometric and physiological variables. Additionally, after the phytoremediation process, pH and OM increased, which indicates that phytotechnology with this plant can improve the conditions of degraded soils contaminated by mining activities. This strengthens the purpose of implementing phytoremediation techniques with wild or native species as a low-cost environmental management strategy that seeks to avoid ecological risks associated with the use of non-native species. Finally, these results contribute to the knowledge of this species about its potential to be considered in ecological restoration or revegetation projects that are associated with phytostabilization to rehabilitate degraded soils contaminated with PTEs. We confirmed that *C. sericea* could be employed as a species for remediation of polluted soils because of its tolerance and accumulation pattern of PTEs, demonstrating the potential to the phytoremediation area. Future research needs to focus on the application of this species under field trials. As a plant that grows naturally in contaminated soils near gold mining areas, there could be different mechanisms and adaptations that promote metal accumulation and plant resistance, leading to better potential results in the remediation process compared to the pot assay. Other research topics to addressed are the study of the cellular compartmentalization of PTEs, and the microorganisms associated with the rhizosphere of this species, which are involved in the absorption of these toxic elements.

## Figures and Tables

**Table 1:** Physicochemical parameters of soils before and after the phytoremediation process.

Treatment	Conditions	Soil Properties					Texture			
		pH (1:1)	OM (%)	S (mg kg^−1^)	P (mg kg^−1^)	CEC (cmol kg^−1^)	Sand (%)	Clay (%)	Silt (%)	Class
T0	Before	5.49 ± 0.15 a	1.26 ± 0.05 a	21 ± 0.26 a	85.2 ± 4.99 a	19.53 ± 0.12 a	26.5	35.6	41.2	CL
	After	5.86 ± 0.05 b	5.29 ± 0.09 b	150.3 ± 7.50 b	104.97 ± 3.57 b	21.23 ± 0.14 b				
T1	Before	5.71 ± 0.01 a	1.0 ± 0.2 a	23.8 ± 2.1 a	102.9 ± 4.5 a	16.33 ± 2.08 a	24.2	32.8	43.1	CL
	After	6.31 ± 0.10 b	3.96 ± 1.36 b	147.3 ± 28.6 b	89.27 ± 9.33 b	19.7 ± 0.12 a				
T2	Before	4.16 ± 0.3 a	0.97 ± 0.1 a	607.4 ± 10.1 a	7.2 ± 0.5 a	11.7 ± 1.4 a	36.7	32.8	30.6	CL
	After	4.17 ± 0.01 a	1.21 ± 0.18 b	850.83 ± 55.9 b	7.43 ± 0.42 a	16.7 ± 0.78 b				
T3	Before	3.54 ± 0.1 a	0.25 ± 0.03 a	726.8 ± 13.4 a	3.9 ± 0.2 a	2.9 ± 0.4 a	29.2	37.8	33.1	CL
	After	4.01 ± 0.15 b	1.21 ± 0.40 b	910.5 ± 36.6 b	32.85 ± 3.85 b	12.6 ± 0.3 b				

Conditions: Before and after the phytoremediation process. CL: Clay loam. Different letters indicate significant statistical differences (*p* < 0.05) using Tukey’s test.

**Table 2:** Concentration and bioavailability of Hg, Pb, and Cd in soils before and after phytoremediation process.

Treatment	Conditions	PTEs (mg kg^−1^)			B.PTEs (%)		
		Hg	Pb	Cd	Hg	Pb	Cd
T0	Before	0.06 ± 0.001 a	2.03 ± 0.08 a	0.52 ± 0.001 a	8.77 ± 1.75 a	0.56 ± 0.03 a	21.24 ± 1.93 a
	After	0.04 ± 0.001 b	1.83 ± 0.05 b	0.45 ± 0.006 b	1.21 ± 0.131 b	0.55 ± 0.02 a	27.47 ± 2.94 a
T1	Before	0.34 ± 0.02 a	8.8 ± 0.18 a	0.81 ± 0.01 a	3.38 ± 0.1 a	0.14 ± 0.01 a	14.24 ± 0.23 a
	After	0.11 ± 0.02 b	7.69 ± 0.64 b	0.72 ± 0.02 b	0.75 ± 0.92 b	0.16 ± 0.01 a	48.36 ± 34.31 b
T2	Before	1.03 ± 0.23 a	134.44 ± 8.05 a	3.4 ± 0.15 a	0.02 ± 0.00 b	0.08 ± 0.01 a	24.59 ± 0.18 a
	After	0.71 ± 0.02 b	93.21 ± 4.75 b	1.78 ± 0.11 b	0.05 ± 0.02 a	0.02 ± 0.01 b	46.18 ± 4.10 b
T3	Before	1.95 ± 0.19 a	178.7 ± 11.2 a	12.7 ± 0.43 a	2.90 ± 0.1 a	0.18 ± 0.03 a	4.96 ± 0.2 a
	After	1.59 ± 0.16 b	120.91 ± 10.63 b	6.45 ± 0.21 b	0.14 ± 0.07 b	0.02 ± 0.02 b	18.21 ± 2.41 b

B.PTEs: Percentage of bioavailability of PTEs. Conditions: Before and after the phytoremediation process. Different letters indicate significant statistical differences (*p* < 0.05) using Tukey’s test.

**Table 3:** Pearson correlation coefficients (*r*-value) between PTEs in soils and in tissues of *C. sericea* and chemical characteristics of soil and PTEs phytoremediation indices (*n* = 12).

PTEs in Soil	PTEs in Plant Tissue					
	Root		Stem		Leaves	
Hg	0.91 ***		0.87 **		0.94 ***	
Pb	0.84 **		0.91 ***		0.69 *	
Cd	0.86 **		0.97 ***		0.82 *	
Chemical Characteristics of Soil	Phytoremediation Indices					
	BCF			TF		
	Hg	Pb	Cd	Hg	Pb	Cd
pH	−0.89 **	−0.12	−0.49	0.88 **	0.92 ***	0.87 **
OM	−0.88 **	−0.41	0.01	0.71 *	0.69 *	0.68 *
S	0.86 **	0.03	0.58*	−0.94 ***	−0.91 ***	−0.88 **
P	−0.80 *	0.00	−0.66*	0.88 **	0.94 ***	0.85 **
CEC	−0.92 ***	−0.26	−0.20	0.85 **	0.77 *	0.77 *

* *p* < 0.05, ** *p* < 0.001, and *** *p* < 0.0001.

## Data Availability

Raw data that support the findings of this study available on request.

## Supplementary Materials

The following supporting information can be downloaded at: https://www.mdpi.com/article/10.3390/plants11050597/s1. Figure S1. *C. sericea* plants in the greenhouse under PTEs stress for 12 weeks. T0: Control treatment; T1: Treatment with low concentration of Hg, Pb, and Cd; T2: Treatment with medium concentration of Hg, Pb, and Cd; T3: Treatment with high concentration of Hg, Pb, and Cd. Figure S2. Phytotoxic symptoms. (a) Leaf necrosis. (b) Fallen leaves. Figure S3. Representation of experimental unit. Table S1. Biomass and photosynthetic pigments of the treatments. Table S2. Certified values and percent recovery for Hg, Pb, and Cd in soil and Lichen.

## References

[1] Cristaldi A., Conti G.O., Jho E.H., Zuccarello P., Grasso A., Copat C., Ferrante M. (2017). Phytoremediation of contaminated soils by heavy metals and PAHs. A brief review. Environ. Technol. Innov..

[2] Khalid S., Shahid M., Niazi N.K., Murtaza B., Bibi I., Dumat C. (2017). A comparison of technologies for remediation of heavy metal contaminated soils. J. Geochem. Explor..

[3] He Z., Shentu J., Yang X., Baligar V., Zhang T., Stoffella P. Heavy Metal Contamination of Soils: Sources, Indicators and Assessment. Proceedings of the 21st International Conference Environmental Indicators.

[4] Marrugo-Negrete J., Durango-Hernández J., Pinedo-Hernández J., Olivero-Verbel J., Díez S. (2015). Phytoremediation of mercury-contaminated soils by Jatropha curcas. Chemosphere.

[5] Marrugo-Negrete J., Marrugo-Madrid S., Pinedo-Hernández J., Durango-Hernández J., Díez S. (2016). Screening of native plant species for phytoremediation potential at a Hg-contaminated mining site. Sci. Total. Environ..

[6] Xiao R., Wang S., Li R., Wang J.J., Zhang Z. (2017). Soil heavy metal contamination and health risks associated with artisanal gold mining in Tongguan, Shaanxi, China. Ecotoxicol. Environ. Saf..

[7] Sako A., Semdé S., Wenmenga U. (2018). Geochemical evaluation of soil, surface water and groundwater around the Tongon gold mining area, northern Côte d’Ivoire, West Africa. J. Afr. Earth Sci..

[8] Gyamfi E., Appiah-Adjei E.K., Adjei K.A. (2019). Potential heavy metal pollution of soil and water resources from artisanal mining in Kokoteasua, Ghana. Groundw. Sustain. Dev..

[9] Adewumi A.J., Laniyan T.A. (2020). Contamination, sources and risk assessments of metals in media from Anka artisanal gold mining area, Northwest Nigeria. Sci. Total Environ..

[10] Kahangwa C.A., Nahonyo C.L., Sangu G., Nassary E.K. (2021). Assessing phytoremediation potentials of selected plant species in restoration of environments contaminated by heavy metals in gold mining areas of Tanzania. Heliyon.

[11] Marrugo-Madrid S., Turull M., Montes G.E., Pico M.V., Marrugo-Negrete J.L., Díez S., Saxena G., Kumar V., Shah M.P. (2021). Chapter 7—Phytoremediation of Mercury in Soils Impacted by Gold Mining: A Case-Study of Colombia. Bioremediation for Environmental Sustainability.

[12] Abdul-Wahab S., Marikar F. (2012). The environmental impact of gold mines: Pollution by heavy metals. Open Eng..

[13] Liu L., Li W., Song W., Guo M. (2018). Remediation techniques for heavy metal-contaminated soils: Principles and applicability. Sci. Total Environ..

[14] Valentim dos Santos J., Varón-López M., Fonsêca Sousa Soares C.R., Lopes Leal P., Siqueira J.O., de Souza Moreira F.M. (2016). Biological attributes of rehabilitated soils contaminated with heavy metals. Environ. Sci. Pollut. Res..

[15] Candeias C., Ávila P., Coelho P., Teixeira J.P., Nriagu J. (2019). Mining Activities: Health Impacts. Encyclopedia of Environmental Health.

[16] Saleem M.H., Ali S., Rehman M., Rana M.S., Rizwan M., Kamran M., Imran M., Riaz M., Soliman M.H., Elkelish E.A. (2020). Influence of phosphorus on copper phytoextraction via modulating cellular organelles in two jute (*Corchorus capsularis* L.) varieties grown in a copper mining soil of Hubei Province, China. Chemosphere.

[17] Sarwar N., Imran M., Shaheen M.R., Ishaque W., Kamran M.A., Matloob A., Rehim A., Hussain S. (2017). Phytoremediation strategies for soils contaminated with heavy metals: Modifications and future perspectives. Chemosphere.

[18] Sabir A., Naveed M., Bashir M.A., Hussain A., Mustafa A., Zahir Z.A., Kamran M., Ditta A., Núñez-Delgado A., Saeed Q. (2020). Cadmium mediated phytotoxic impacts in Brassica napus: Managing growth, physiological and oxidative disturbances through combined use of biochar and Enterobacter sp. MN17. J. Environ. Manag..

[19] Wan X., Lei M., Chen T. (2016). Cost-benefit calculation of phytoremediation technology for heavy-metal-contaminated soil. Sci. Total Environ..

[20] Mahar A., Wang P., Ali A., Awasthi M.K., Lahori A.H., Wang Q., Li R., Zhang Z. (2016). Challenges and opportunities in the phytoremediation of heavy metals contaminated soils: A review. Ecotoxicol. Environ. Saf..

[21] Murakami M., Ae N., Ishikawa S. (2007). Phytoextraction of cadmium by rice (*Oryza sativa* L.), soybean (*Glycine max* (L.) Merr.), and maize (*Zea mays* L.). Environ. Pollut..

[22] Salas-Moreno M., Marrugo-Negrete J. (2020). Phytoremediation potential of Cd and Pb-contaminated soils by Paspalum fasciculatum Willd. ex Flüggé. Int. J. Phytoremediat..

[23] Antoniadis V., Shaheen S.M., Stärk H.-J., Wennrich R., Levizou E., Merbach I., Rinklebe J. (2021). Phytoremediation potential of twelve wild plant species for toxic elements in a contaminated soil. Environ. Int..

[24] Lum A.F., Ngwa E.S.A., Chikoye D., Suh C.E. (2014). Phytoremediation Potential of Weeds in Heavy Metal Contaminated Soils of the Bassa Industrial Zone of Douala, Cameroon. Int. J. Phytoremediat..

[25] Cheng S.-F., Huang C.-Y., Chen K.-L., Lin S.-C., Lin Y.-C. (2016). Phytoattenuation of lead-contaminated agricultural land using Miscanthus floridulus—An in situ case study. Desalination Water Treat..

[26] Xu L., Xing X., Liang J., Peng J., Zhou J. (2019). In situ phytoremediation of copper and cadmium in a co-contaminated soil and its biological and physical effects. RSC Adv..

[27] Zhang L., Zhang P., Yoza B., Liu W., Liang H. (2020). Phytoremediation of metal-contaminated rare-earth mining sites using Paspalum conjugatum. Chemosphere.

[28] Murakami M., Ae N. (2009). Potential for phytoextraction of copper, lead, and zinc by rice (*Oryza sativa* L.), soybean (*Glycine max* [L.] Merr.), and maize (*Zea mays* L.). J. Hazard. Mater..

[29] Ibaraki T., Kuroyanagi N., Murakami M. (2009). Practical phytoextraction in cadmium-polluted paddy fields using a high cadmium accumulating rice plant cultured by early drainage of irrigation water. Soil Sci. Plant Nutr..

[30] Willscher S., Mirgorodsky D., Jablonski L., Ollivier D., Merten D., Büchel G., Wittig J., Werner P. (2013). Field scale phytoremediation experiments on a heavy metal and uranium contaminated site, and further utilization of the plant residues. Hydrometallurgy.

[31] Takahashi R., Ito M., Katou K., Sato K., Nakagawa S., Tezuka K., Akagi H., Kawamoto T. (2016). Breeding and characterization of the rice (*Oryza sativa* L.) line “Akita 110” for cadmium phytoremediation. Soil Sci. Plant Nutr..

[32] Luo J., He M., Qi S., Wu J., Gu X.S. (2018). Effect of planting density and harvest protocol on field-scale phytoremediation efficiency by Eucalyptus globulus. Environ. Sci. Pollut. Res..

[33] Tosini L., Folzer H., Heckenroth A., Prudent P., Santonja M., Farnet A.-M., Salducci M.-D., Vassalo L., Labrousse Y., Oursel B. (2020). Gain in biodiversity but not in phytostabilization after 3 years of ecological restoration of contaminated Mediterranean soils. Ecol. Eng..

[34] Rocha Martins W., Douglas Roque Lima M., de Oliveira Barros Junior U., Sousa Villas-Boas Amorim L., de Assis Oliveira F., Schwartz G. (2020). Ecological methods and indicators for recovering and monitoring ecosystems after mining: A global literature review. Ecol. Eng..

[35] Suchkova N., Tsiripidis I., Alifragkis D., Ganoulis J., Darakas E., Sawidis T.H. (2014). Assessment of phytoremediation potential of native plants during the reclamation of an area affected by sewage sludge. Ecol. Eng..

[36] Matanzas N., Afif E., Díaz T.E., Gallego J.R. (2021). Phytoremediation Potential of Native Herbaceous Plant Species Growing on a Paradigmatic Brownfield Site. Water Air Soil Pollut..

[37] Bahrami M., Jahantab E., Mahmoudi M.R. (2021). Clustering the organic soil amendments in combination with phytoremediation of heavy metals contaminated soil. Int. J. Environ. Anal. Chem..

[38] Futughe A.E., Purchase D., Jones H., Shmaefsky B.R. (2020). Phytoremediation Using Native Plants. Phytoremediation: In-Situ Applications.

[39] Wu B., Peng H., Sheng M., Luo H., Wang X., Zhang R., Xu F., Xu H. (2021). Evaluation of phytoremediation potential of native dominant plants and spatial distribution of heavy metals in abandoned mining area in Southwest China. Ecotoxicol. Environ. Saf..

[40] Fernández S., Poschenrieder C., Marcenò C., Gallego J.R., Jiménez-Gámez D., Bueno A., Afif E. (2017). Phytoremediation capability of native plant species living on Pb-Zn and Hg-As mining wastes in the Cantabrian range, north of Spain. J. Geochem. Explor..

[41] Bernal R., Galeano G., Rodríguez A., Sarmiento H., Gutiérrez M. Mortiño (Clidemia Sericea—Melastomatáceas). Nombres Comunes de las Plantas de Colombia. http://www.biovirtual.unal.edu.co/nombrescomunes/es/detalle/ncientifico/22693/.

[42] Chamba I., Gazquez M.J., Selvaraj T., Calva J., Toledo J.J., Armijos C. (2016). Selection of a suitable plant for phytoremediation in mining artisanal zones. Int. J. Phytoremediat..

[43] Combatt E.M.C., Palencia G., Marin N. (2003). Classification of acid sulphate soils for the Extractable sulphur in the municipalities of the low Sinu valley in the department of Cordoba. Temas Agrar..

[44] Kim K.-R., Owens G., Kwon S. (2010). Influence of Indian mustard (*Brassica juncea*) on rhizosphere soil solution chemistry in long-term contaminated soils: A rhizobox study. J. Environ. Sci..

[45] Luo Y.M., Christie P., Baker A.J.M. (2000). Soil solution Zn and pH dynamics in non-rhizosphere soil and in the rhizosphere of Thlaspi caerulescens grown in a Zn/Cd-contaminated soil. Chemosphere.

[46] Rosenfeld C.E., Chaney R.L., Martínez C.E. (2018). Soil geochemical factors regulate Cd accumulation by metal hyperaccumulating Noccaea caerulescens (J. Presl & C. Presl) F.K. Mey in field-contaminated soils. Sci. Total Environ..

[47] Kumar R., Pandey S., Pandey A. (2006). Plant roots and carbon sequestration. Curr. Sci..

[48] Antoniadis V., Levizou E., Shaheen S.M., Ok Y.S., Sebastian A., Baum C., Prasad M.N., Wenzel W.W., Rinklebe J. (2017). Trace elements in the soil-plant interface: Phytoavailability, translocation, and phytoremediation–A review. Earth-Sci. Rev..

[49] Gomes P., Valente T., Braga M.A.S., Grande J.A., de la Torre M.L. (2016). Enrichment of trace elements in the clay size fraction of mining soils. Environ. Sci. Pollut. Res. Int..

[50] Čížková B., Woś B., Pietrzykowski M., Frouz J. (2018). Development of soil chemical and microbial properties in reclaimed and unreclaimed grasslands in heaps after opencast lignite mining. Ecol. Eng..

[51] Yin D., He T., Yin R., Zeng L. (2018). Effects of soil properties on production and bioaccumulation of methylmercury in rice paddies at a mercury mining area, China. J. Environ. Sci..

[52] Boldt-Burisch K., Schneider B.U., Naeth M.A., Hüttl R.F. (2019). Root Exudation of Organic Acids of Herbaceous Pioneer Plants and Their Growth in Sterile and Non-Sterile Nutrient-Poor, Sandy Soils from Post-Mining Sites. Pedosphere.

[53] Marrugo-Negrete J., Pinedo-Hernández J., Díez S. (2017). Assessment of heavy metal pollution, spatial distribution and origin in agricultural soils along the Sinú River Basin, Colombia. Environ. Res..

[54] Raj D., Kumar A., Maiti S.K. (2020). Mercury remediation potential of *Brassica juncea* (L.) Czern. for clean-up of flyash contaminated sites. Chemosphere.

[55] Oseni O.M., Dada O.E., Okunlola G.O., Ajao A.A. (2018). Phytoremediation Potential of *Chromolaena odorata* (L.) King and Robinson (*Asteraceae*) and *Sida acuta* Burm. f. (*Malvaceae*) Grown in lead-Polluted Soils. Jordan J. Biol. Sci..

[56] Koopmans G.F., Römkens P.F.A.M., Fokkema M.J., Song J., Luo Y.M., Japenga J., Zhao F. (2008). Feasibility of phytoextraction to remediate cadmium and zinc contaminated soils. Environ. Pollut..

[57] Kubota H., Sugawara R., Kitajima N., Yajima S., Tani S. (2010). Cadmium phytoremediation by Arabidopsis halleri ssp. gemmifera. Jpn. J. Soil Sci. Plant Nutr..

[58] Yang Y., Ge Y., Zeng H., Zhou X., Peng L., Zeng Q. (2017). Phytoextraction of cadmium-contaminated soil and potential of regenerated tobacco biomass for recovery of cadmium. Sci. Rep..

[59] Li Q., Ji H., Qin F., Tang L., Guo X., Feng J. (2014). Sources and the distribution of heavy metals in the particle size of soil polluted by gold mining upstream of Miyun Reservoir, Beijing: Implications for assessing the potential risks. Environ. Monit. Assess..

[60] Velásquez Ramírez M.G., Barrantes J.A.G., Thomas E., Gamarra Miranda L.A., Pillaca M., Tello Peramas L.D., Tapia L.R.B. (2020). Heavy metals in alluvial gold mine spoils in the peruvian amazon. CATENA.

[61] Ogundele L.T., Oluwajana O.A., Ogunyele A.C., Inuyomi S.O. (2021). Heavy metals, radionuclides activity and mineralogy of soil samples from an artisanal gold mining site in Ile-Ife, Nigeria: Implications on human and environmental health. Environ. Earth Sci..

[62] Kaninga B.K., Chishala B.H., Maseka K.K., Sakala G.M., Lark M.R., Tye A., Watts M.J. (2020). Review: Mine tailings in an African tropical environment—mechanisms for the bioavailability of heavy metals in soils. Environ. Geochem. Health.

[63] Shahid M., Dumat C., Khalid S., Niazi N.K., Antunes P.M.C., De Voogt P., Gunther F.A. (2017). Cadmium Bioavailability, Uptake, Toxicity and Detoxification in Soil-Plant System. Reviews of Environmental Contamination and Toxicology Volume 241.

[64] Romeh A.A., Khamis M.A., Metwally S.M. (2015). Potential of *Plantago major* L. for Phytoremediation of Lead-Contaminated Soil and Water. Water Air Soil Pollut..

[65] Zhao X., He B., Wu H., Zheng G., Ma X., Liang J., Li P., Fan Q. (2020). A comprehensive investigation of hazardous elements contamination in mining and smelting-impacted soils and sediments. Ecotoxicol. Environ. Saf..

[66] Li Y., Zhao J., Guo J., Liu M., Xu Q., Li H., Li Y.F., Zheng L., Zhang Z., Gao Y. (2017). Influence of sulfur on the accumulation of mercury in rice plant (*Oryza sativa* L.) growing in mercury contaminated soils. Chemosphere.

[67] Zhang Z., Cao Y., Li J., Cai C., Huang Z. (2014). Spatial distribution and bioavailability of Hg in vegetable-growing soils collected from the estuary areas of Jiulong River, China. Environ. Earth Sci..

[68] Li J., Li K., Cave M., Li H.-B., Ma L.Q. (2015). Lead bioaccessibility in 12 contaminated soils from China: Correlation to lead relative bioavailability and lead in different fractions. J. Hazard. Mater..

[69] Kasemodel M.C., Lima J.Z., Sakamoto I.K., Varesche M.B.A., Trofino J.C., Rodrigues V.G.S. (2016). Soil contamination assessment for Pb, Zn and Cd in a slag disposal area using the integration of geochemical and microbiological data. Environ. Monit. Assess..

[70] Różański S.Ł., Castejón J.M.P., Fernández G.G. (2016). Bioavailability and mobility of mercury in selected soil profiles. Environ. Earth Sci..

[71] Zhang J., Li H., Zhou Y., Dou L., Cai L., Mo L., You J. (2018). Bioavailability and soil-to-crop transfer of heavy metals in farmland soils: A case study in the Pearl River Delta, South China. Environ. Pollut..

[72] Pang J., Han J., Fan X., Li C., Dong X., Liang L., Chen Z. (2019). Mercury speciation, bioavailability and risk assessment on soil–rice systems from a watershed impacted by abandoned Hg mine-waste tailings. Acta Geochim..

[73] Liang L., Xu X., Han J., Xu Z., Wu P., Guo J., Qiu G. (2019). Characteristics, speciation, and bioavailability of mercury and methylmercury impacted by an abandoned coal gangue in southwestern China. Environ. Sci. Pollut. Res..

[74] Marrugo-Negrete J., Pinedo-Hernández J., Combatt E.M., Bravo A.G., Díez S. (2019). Flood-induced metal contamination in the topsoil of floodplain agricultural soils: A case-study in Colombia. Land Degrad. Dev..

[75] Li X., Zhang X., Wang X., Cui Z. (2019). Phytoremediation of multi-metal contaminated mine tailings with *Solanum nigrum* L. and biochar/attapulgite amendments. Ecotoxicol. Environ. Saf..

[76] Zhou Y., Wang L., Xiao T., Chen Y., Beiyuan J., She J., Zhou Y., Yin M., Liu J., Liu Y. (2020). Legacy of multiple heavy metal(loid)s contamination and ecological risks in farmland soils from a historical artisanal zinc smelting area. Sci. Total Environ..

[77] Luo L., Shen Y., Wang X., Chu B., Xu T., Liu Y., Zeng Y., Liu J. (2018). Phytoavailability, bioaccumulation, and human health risks of metal(loid) elements in an agroecosystem near a lead-zinc mine. Environ. Sci. Pollut. Res..

[78] Gautam M., Pandey D., Agrawal S.B., Agrawal M., Singh A., Prasad S.M., Singh R.P. (2016). Metals from Mining and Metallurgical Industries and Their Toxicological Impacts on Plants. Plant Responses to Xenobiotics.

[79] Kalaivanan D., Ganeshamurthy A.N., Rao N.K.S., Shivashankara K.S., Laxman R.H. (2016). Mechanisms of Heavy Metal Toxicity in Plants. Abiotic Stress Physiology of Horticultural Crops.

[80] Pajević S., Borišev M., Nikolić N., Arsenov D.D., Orlović S., Župunski M., Ansari A.A., Gill S.S., Gill R., Lanza G.R., Newman L. (2016). Phytoextraction of Heavy Metals by Fast-Growing Trees: A Review. Phytoremediation: Management of Environmental Contaminants, Volume 3.

[81] Gorelova S.V., Frontasyeva M.V., Ansari A.A., Gill S.S., Gill R.R., Lanza G., Newman L. (2017). The Use of Higher Plants in Biomonitoring and Environmental Bioremediation. Phytoremediation: Management of Environmental Contaminants, Volume 5.

[82] Marrugo-Negrete J., Durango-Hernández J., Pinedo-Hernández J., Enamorado-Montes G., Díez S. (2016). Mercury uptake and effects on growth in Jatropha curcas. J. Environ. Sci..

[83] Wu M., Luo Q., Liu S., Zhao Y., Long Y., Pan Y. (2018). Screening ornamental plants to identify potential Cd hyperaccumulators for bioremediation. Ecotoxicol. Environ. Saf..

[84] Cheng S. (2003). Effects of Heavy metals on plants and resistance mechanisms. Environ. Sci. Pollut. Res..

[85] Kumar B., Smita K., Cumbal Flores L. (2017). Plant mediated detoxification of mercury and lead. Arab. J. Chem..

[86] Zhou J., Li Z., Zhou T., Xin Z., Wu L., Luo Y., Christie P. (2020). Aluminum toxicity decreases the phytoextraction capability by cadmium/zinc hyperaccumulator Sedum plumbizincicola in acid soils. Sci. Total Environ..

[87] Montaño Santana J.C., Forero Ulloa F.E. (2013). The effect of organic byproducts of the jaggery production process on the physical properties of a sulfate acid soil. Cienc Tecnol. Agropecu..

[88] Bernal A.A., Montaño J.C., Sánchez R., Albarrán Y.L., Forero F.E. (2017). Evaluation of Organic Materials and Liming on Exchangeable Bases of an Acid Sulphate Soil at Greenhouse Conditions. https://repositorio.unicordoba.edu.co/handle/ucordoba/355.

[89] Ding W., Zhang J., Wu S.-C., Zhang S., Christie P., Liang P. (2019). Responses of the grass *Paspalum distichum* L. to Hg stress: A proteomic study. Ecotoxicol. Environ. Saf..

[90] Huang Y., Xi Y., Gan L., Johnson D., Wu Y., Ren D., Liu H. (2019). Effects of lead and cadmium on photosynthesis in Amaranthus spinosus and assessment of phytoremediation potential. Int. J. Phytoremediat..

[91] Xu J., Zhang J., Lv Y., Xu K., Lu S., Liu X., Yang Y. (2020). Effect of soil mercury pollution on ginger (*Zingiber officinale Roscoe*): Growth, product quality, health risks and silicon mitigation. Ecotoxicol. Environ. Saf..

[92] Gonçalves A.C., Schwantes D., Braga de Sousa R.F., Benetoli da Silva T.R., Guimarães V.F., Campagnolo M.A., de Vasconcelos E.S., Zimmermann J. (2020). Phytoremediation capacity, growth and physiological responses of Crambe abyssinica Hochst on soil contaminated with Cd and Pb. J. Environ. Manage..

[93] Dinu C., Vasile G.-G., Buleandra M., Popa D.E., Gheorghe S., Ungureanu E.-M. (2020). Translocation and accumulation of heavy metals in *Ocimum basilicum* L. plants grown in a mining-contaminated soil. J. Soils Sediments.

[94] Ouzounidou G., Moustakas M., Eleftheriou E.P. (1997). Physiological and Ultrastructural Effects of Cadmium on Wheat (*Triticum aestivum* L.) Leaves. Arch. Environ. Contam. Toxicol..

[95] Liu D., Li T.-Q., Jin X.-F., Yang X.-E., Islam E., Mahmood Q. (2008). Lead Induced Changes in the Growth and Antioxidant Metabolism of the Lead Accumulating and Non-accumulating Ecotypes of Sedum alfredii. J. Integr. Plant Biol..

[96] Huihui Z., Xin L., Zisong X., Yue W., Zhiyuan T., Meijun A., Yuehui Z., Wenxu Z., Nan X., Guangyu S. (2020). Toxic effects of heavy metals Pb and Cd on mulberry (*Morus alba* L.) seedling leaves: Photosynthetic function and reactive oxygen species (ROS) metabolism responses. Ecotoxicol. Environ. Saf..

[97] Mani D., Kumar C., Patel N.K. (2015). Hyperaccumulator Oilcake Manure as an Alternative for Chelate-Induced Phytoremediation of Heavy Metals Contaminated Alluvial Soils. Int. J. Phytoremediat..

[98] Małkowski E., Sitko K., Zieleźnik-Rusinowska P., Gieroń Ż., Szopiński M., Sablok G. (2019). Heavy Metal Toxicity: Physiological Implications of Metal Toxicity in Plants. Plant Metallomics and Functional Omics: A System-Wide Perspective.

[99] Cui X., Mao P., Sun S., Huang R., Fan Y., Li Y., Zhuang P., Li Z. (2021). Phytoremediation of cadmium contaminated soils by *Amaranthus Hypochondriacus* L.: The effects of soil properties highlighting cation exchange capacity. Chemosphere.

[100] Patra M., Sharma A. (2000). Mercury toxicity in plants. Bot. Rev..

[101] Amari T., Ghnaya T., Abdelly C. (2017). Nickel, cadmium and lead phytotoxicity and potential of halophytic plants in heavy metal extraction. S. Afr. J. Bot..

[102] Nisar N., Li L., Lu S., Khin N.C., Pogson B.J. (2015). Carotenoid Metabolism in Plants. Mol. Plant.

[103] Devi Prasad P.V., Devi Prasad P.S. (1982). Effect of cadmium, lead and nickel on three freshwater green algae. Water Air Soil Pollut. Neth..

[104] Puzon J.J.M., Rivero G.C., Serrano J.E. (2014). Antioxidant responses in the leaves of mercury-treated Eichhornia crassipes (Mart.) Solms. Environ. Monit. Assess..

[105] Ozyigit I.I., Dogan I., Igdelioglu S., Filiz E., Karadeniz S., Uzunova Z. (2016). Screening of damage induced by lead (Pb) in rye (*Secale cereale* L.)—A genetic and physiological approach. Biotechnol. Biotechnol. Equip..

[106] Chandra R., Kang H. (2016). Mixed heavy metal stress on photosynthesis, transpiration rate, and chlorophyll content in poplar hybrids. For. Sci Technol..

[107] Fargašová A., Molnárová M. (2010). Assessment of Cr and Ni phytotoxicity from cutlery-washing waste-waters using biomass and chlorophyll production tests on mustard *Sinapis alba* L. seedlings. Environ. Sci. Pollut. Res..

[108] Chinmayee M.D., Mahesh B., Pradesh S., Mini I., Swapna T.S. (2012). The Assessment of Phytoremediation Potential of Invasive Weed *Amaranthus spinosus* L. Appl. Biochem. Biotechnol..

[109] Leal-Alvarado D.A., Espadas-Gil F., Sáenz-Carbonell L., Talavera-May C., Santamaría J.M. (2016). Lead accumulation reduces photosynthesis in the lead hyper-accumulator Salvinia minima Baker by affecting the cell membrane and inducing stomatal closure. Aquat. Toxicol..

[110] Zhang H., Xu Z., Guo K., Huo Y., He G., Sun H., Guan Y., Xu N., Yang W., Sun G. (2020). Toxic effects of heavy metal Cd and Zn on chlorophyll, carotenoid metabolism and photosynthetic function in tobacco leaves revealed by physiological and proteomics analysis. Ecotoxicol. Environ. Saf..

[111] Nasser S., Soad E., Fatma E.-S. (2014). Phytoremediation of Lead and Cadmium Contaminated Soils Using Sunflower Plant. J. Stress Physiol. Amp. Biochem..

[112] Marrugo-Negrete J., Durango-Hernández J., Díaz-Fernández L., Urango-Cárdenas I., Araméndiz-Tatis H., Vergara-Flórez V., Bravo A.G., Díez S. (2020). Transfer and bioaccumulation of mercury from soil in cowpea in gold mining sites. Chemosphere.

[113] Kumar A., Aery N.C., Singh A., Prasad S.M., Singh R.P. (2016). Impact, Metabolism, and Toxicity of Heavy Metals in Plants. Plant Responses to Xenobiotics.

[114] Saghi A., Rashed Mohassel M.H., Parsa M., Hammami H. (2016). Phytoremediation of lead-contaminated soil by Sinapis arvensis and Rapistrum rugosum. Int. J. Phytoremediat..

[115] Shaik J., Sumithra S., Senthilkumar P. (2018). Mercury uptake and translocation by indigenous plants. Rasayan J. Chem..

[116] Ghori N.-H., Ghori T., Hayat M.Q., Imadi S.R., Gul A., Altay V., Ozturk M. (2019). Heavy metal stress and responses in plants. Int. J. Environ. Sci. Technol..

[117] Herlina L., Widianarko B., Purnaweni H., Sudarno S., Sunoko H.R. (2020). Phytoremediation of Lead Contaminated Soil Using Croton (*Cordiaeumvariegatum*) Plants. J. Ecol. Eng..

[118] Fu X., Dou C., Chen Y., Chen X., Shi J., Yu M., Xu J. (2011). Subcellular distribution and chemical forms of cadmium in *Phytolacca americana* L. J. Hazard. Mater..

[119] Chen Z., Zhao Y., Fan L., Xing L., Yang Y. (2015). Cadmium (Cd) Localization in Tissues of Cotton (*Gossypium hirsutum* L.), and Its Phytoremediation Potential for Cd-Contaminated Soils. Bull. Environ. Contam. Toxicol..

[120] Nacke H., Gonçalves A.C., Schwantes D., Nava I.A., Strey L., Coelho G.F. (2013). Availability of Heavy Metals (Cd, Pb, and Cr) in Agriculture from Commercial Fertilizers. Arch. Environ. Contam. Toxicol..

[121] Concas S., Lattanzi P., Bacchetta G., Barbafieri M., Vacca A. (2015). Zn, Pb and Hg Contents of *Pistacia lentiscus* L. Grown on Heavy Metal-Rich Soils: Implications for Phytostabilization. Water Air Soil Pollut..

[122] Nworie O.E., Lin C. (2021). Seasonal variation in tissue-borne heavy Metal(loid)s in herbaceous plants growing in contaminated soils developed from industrial wastes of industrial revolution age. Environ. Adv..

[123] Sharma P., Tripathi S., Chandra R. (2021). Highly efficient phytoremediation potential of metal and metalloids from the pulp paper industry waste employing *Eclipta alba* (L) and *Alternanthera philoxeroide* (L): Biosorption and pollution reduction. Bioresour. Technol..

[124] Khan S., Farooq R., Shahbaz S., Khan M.A., Sadique M. (2009). Health Risk Assessment of Heavy Metals for Population via Consumption of Vegetables. World Appl. Sci. J..

[125] Mousavi Kouhi S.M., Moudi M. (2020). Assessment of phytoremediation potential of native plant species naturally growing in a heavy metal-polluted saline–sodic soil. Environ. Sci. Pollut. Res..

[126] Hasnaoui S.E., Fahr M., Keller C., Levard C., Angeletti B., Chaurand P., Triqui Z.E.A., Guedira A., Rhazi L., Colin F. (2020). Screening of Native Plants Growing on a Pb/Zn Mining Area in Eastern Morocco: Perspectives for Phytoremediation. Plants.

[127] Mitra G.N., Mitra G.N. (2015). Uptake of Heavy Metals. Regulation of Nutrient Uptake by Plants: A Biochemical and Molecular Approach.

[128] Zheng Y., Shen D., Wu S., Han Y., Li S., Tang F., Ni Z., Mo R., Liu Y. (2018). Uptake effects of toxic heavy metals from growth soils into jujube and persimmon of China. Environ. Sci. Pollut. Res..

[129] Boening D.W. (2000). Ecological effects, transport, and fate of mercury: A general review. Chemosphere.

[130] Galal T.M., Gharib F.A., Ghazi S.M., Mansour K.H. (2017). Metal uptake capability of *Cyperus articulatus* L. and its role in mitigating heavy metals from contaminated wetlands. Environ. Sci. Pollut. Res. Int..

[131] Do Nascimento Júnior A.L., de QPaiva A., da Souza L., Souza-Filho L.F., Souza L.D., Fernandes Filho E.I., Schaefer C.E.R.G., da Silva E.F., Fernandes A.C.O., da S. Xavier F.A. (2021). Heavy metals distribution in different parts of cultivated and native plants and their relationship with soil content. Int. J. Environ. Sci. Technol..

[132] Eid E.M., Galal T.M., El-Bebany A.F. (2020). Prediction models for monitoring heavy-metal accumulation by wheat (*Triticum aestivum* L.) plants grown in sewage sludge amended soil. Int. J. Phytoremediat..

[133] Cui Y., Dong Y., Li H., Wang Q. (2004). Effect of elemental sulphur on solubility of soil heavy metals and their uptake by maize. Environ. Int..

[134] Smolinska B., Leszczynska J. (2017). Photosynthetic pigments and peroxidase activity of *Lepidium sativum* L. during assisted Hg phytoextraction. Environ. Sci. Pollut. Res..

[135] Luo J., Cai L., Qi S., Wu J., Sophie Gu X. (2017). A multi-technique phytoremediation approach to purify metals contaminated soil from e-waste recycling site. J. Environ. Manag..

[136] Guo D., Ali A., Ren C., Du J., Li R., Lahori A.H., Xiao R., Zhang Z., Zhang Z. (2019). EDTA and organic acids assisted phytoextraction of Cd and Zn from a smelter contaminated soil by potherb mustard (*Brassica juncea*, *Coss*) and evaluation of its bioindicators. Ecotoxicol. Environ. Saf..

[137] Lichtenthaler H.K. (1987). [34] Chlorophylls and Carotenoids: Pigments of Photosynthetic Biomembranes. Methods in Enzymology.

[138] Day R.P., Black C.A., Evans D.D., White J.L., Ensminger L.E., Clark F.E. (1965). Pipette Method of Particle Size Analysis.

[139] Walkley A. (1947). A critical examination of a rapid method for determining organic carbon in soils—Effect of variations in digestion conditions and of inorganic soil constituents. Soil Sci..

[140] Walkley A., Black I.A. (1934). An examination of the Degtjareff method for determining soil organic matter, and a proposed modification of the chromic acid titration method. Soil Sci..

[141] Fox R.L., Olson R.A., Rhoades H.F. (1964). Evaluating the Sulfur Status of Soils by Plant and Soil Tests. Soil Sci. Soc. Am. J..

[142] Bray R.H., Kurtz L.T. (1945). Determination of total, organic, and available forms of phosphorus in soils. Soil Sci..

[143] Reeuwij L.P. (2002). Procedures for Soil Analysis.

[144] US EPA O. (2007). EPA Method 7473: Mercury in Solids and Solutions by Thermal Decomposition, Amalgamation, and Atomic Absorption Spectrophotometry. https://www.epa.gov/sites/default/files/2015-07/documents/epa-7473.pdf.

[145] US EPA O. (2007). Method 3051A: Microwave Assisted Acid Digestion of Sediments, Sludges, and Oils. https://www.epa.gov/sites/production/files/2015-12/documents/3051a.pdf.

[146] Rauret G., López-Sánchez J.-F., Sahuquillo A., Barahona E., Lachica M., Ure A.M., Davidson C.M., Gomez A., Lück D., Bacon J. (2000). Application of a modified BCR sequential extraction (three-step) procedure for the determination of extractable trace metal contents in a sewage sludge amended soil reference material (CRM 483), complemented by a three-year stability study of acetic acid and EDTA extractable metal content. J. Environ. Monit..

[147] Buccolieri A., Buccolieri G., Cardellicchio N., Dell’Atti A., Di Leo A., Maci A. (2006). Heavy metals in marine sediments of Taranto Gulf (Ionian Sea, Southern Italy). Mar. Chem..

[148] Yoon J., Cao X., Zhou Q., Ma L.Q. (2006). Accumulation of Pb, Cu, and Zn in native plants growing on a contaminated Florida site. Sci. Total Environ..

[149] Yanqun Z., Yuan L., Jianjun C., Haiyan C., Li Q., Schvartz C. (2005). Hyperaccumulation of Pb, Zn and Cd in herbaceous grown on lead–zinc mining area in Yunnan, China. Environ. Int..

