# Supplementary material for: Phytoremediation of Soils Contaminated with Heavy Metals from Gold Mining Activities Using Clidemia sericea D. Don
Source: Plants (Basel). 2022 Feb 23;11(5):597. doi: 10.3390/plants11050597 (PMC8912359; doi:10.3390/plants11050597)
Supplement: Supplementary file 1 [file plants-11-00597-s001.zip › plants-1527187-supplementary.pdf]

PHYTOREMEDIATION OF SOILS CONTAMINATED WITH HEAVY METALS FROM GOLD MINING ACTIVITIES USING *Clidemia sericea D. Don*.

Elvia Valeria Durante-Yánez[1], María Alejandra Martínez-Macea[1], Germán Enamorado-Montes[1], Enrique Combatt Caballero[2] and José Marrugo-Negrete[1*]

[1] Water, Applied, and Environmental Chemistry Research Group, Department of Chemistry, Faculty of Basic Sciences, University of Córdoba, Montería, 230002, Colombia; evdurante@correo.unicordoba.edu.co (E.V.D.-Y.); mariamartinezm@correo.unicordoba.edu.co (M.A.M.-M.); genamoradomontes@correo.unicordoba.edu.co (G.E.-M)

[2] Department of Agricultural Engineering and Rural Development, Faculty of Agricultural Sciences, University of Córdoba, Montería, 230002, Colombia; emcombatt@correo.unicordoba.edu.co (E.C.C)

*** Corresponding author** José Marrugo-Negrete, Water, Applied and Environmental Chemistry Research Group. University of Córdoba, carrera 6 No. 77- 305 Montería - Córdoba, Colombia. Zip Code: 230002. e-mail: jmarrugo@correo.unicordoba.edu.co (J.M.-N).

List of Figures

Figure S1. *C. sericea* plants in the greenhouse under PTEs stress for 12 weeks. T0: Control treatment; T1: Treatment with low concentration of Hg, Pb and Cd; T2: Treatment with medium concentration of Hg, Pb and Cd; T3: Treatment with high concentration of Hg, Pb and Cd.

Figure S2. Phytotoxic symptoms. a) Leaf necrosis. b) Fallen leaves.

Figure S3. Representation of experimental unit.

List of Tables

Table S1. Biomass and photosynthetic pigments of the treatments.

Table S2. Certified values and percent recovery for Hg, Pb and Cd in soil and Lichen.

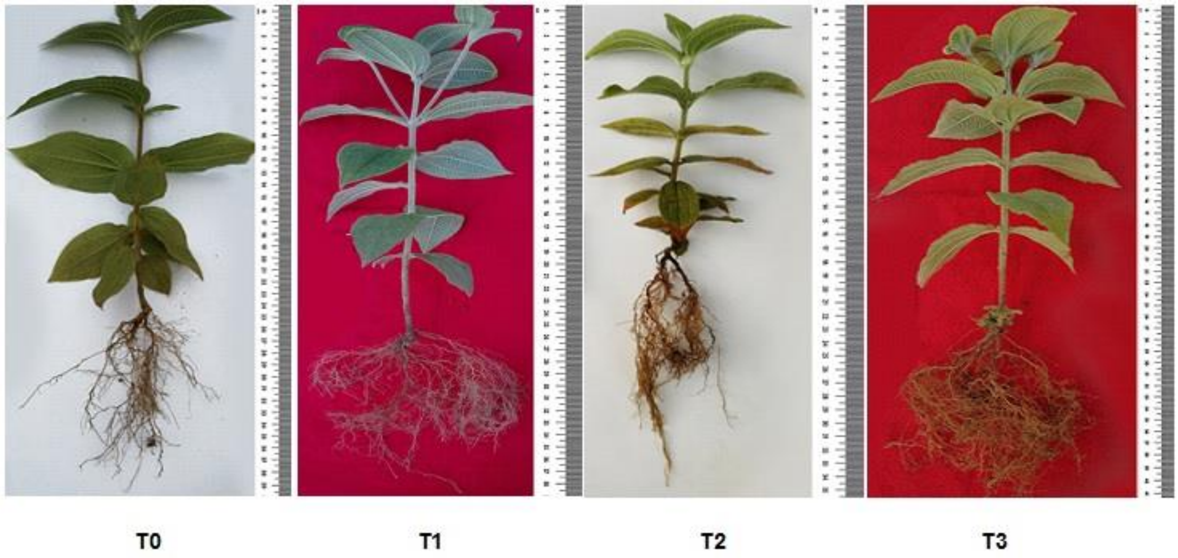

Figure S1. *C. sericea* plants in the greenhouse under PTEs stress for 12 weeks. T0: Control treatment; T1: Treatment with low concentration of Hg, Pb and Cd; T2: Treatment with medium concentration of Hg, Pb and Cd; T3: Treatment with high concentration of Hg, Pb and Cd.

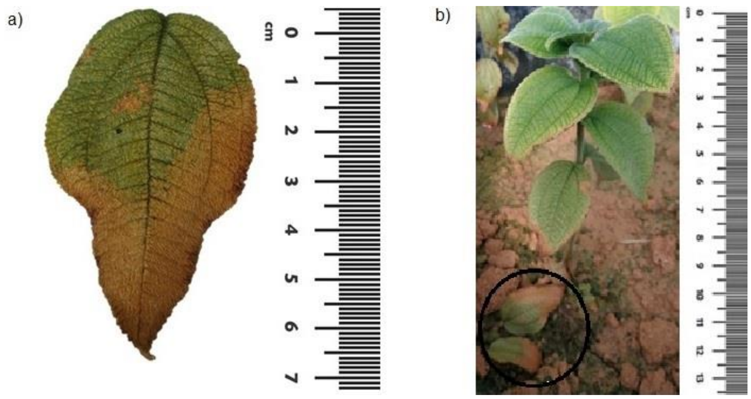

Figure S2. Phytotoxic symptoms. a) Leaf necrosis. b) Fallen leaves.

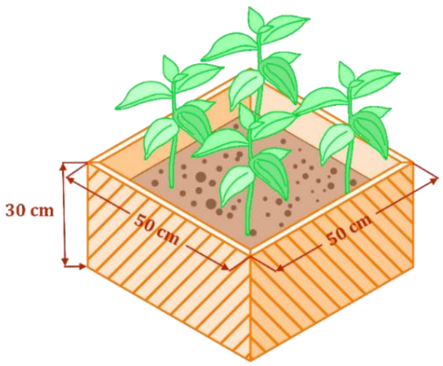

Figure S3. Representation of experimental unit.

Table S1. Biomass and photosynthetic pigments of the treatments.

Variables	Treatments			
**Dry biomass**	**T0**	**T1**	**T2**	**T3**
Total biomass (g)	4.95±0.15a	3.83±0.13ab	2.64±0.92b	2.47±0.63b
Root (g)	1.14±0.09a	0.95±0.11ab	0.6±0.24b	0.66±0.21b
Stem (g)	1.27±0.04a	0.95±0.09a	0.51±0.19b	0.49±0.15b
Leaves (g)	2.54±0.09a	1.92±0.32ab	1.54±0.49b	1.32±0.32b
Necrosed leaves (g)	n.p	n.p	0.13±0.03a	0.12±0.07a
**Photosynthetic pigments**	**T0**	**T1**	**T2**	**T3**
Chlorophyll a (mg g^{-1})	9.86±0.08b	9.90±0.01b	1.36±0.01c	12.29±0.01a
Chlorophyll b (mg g^{-1})	3.85±0.08c	3.96±0.01b	0.53±0.01d	13.47±0.01a
Chlorophyll a + b (mg g^{-1})	13.71±0.16b	13.86±0.01b	1.89±0.01c	25.76±0.02a
Carotenoids (mg g^{-1})	3.18±0.05a	3.01±0.02b	0.56±0.01d	1.53±0.02c

n.p: did not present. Total biomass does not include necrotic leaves because they were collected before the end of the trial. Different letters indicate significant statistical differences ($p<0.05$) using Tukey's test between each treatment per variable.

Table S2. Certified values and percent recovery for Hg, Pb and Cd in soil and Lichen.

Metal	Certified reference material	Matrix	Certified Value (mg kg^{-1})	Recovery percentage (%)
Hg	CRM008–050	Soil	0.720± 0.03	95.8
	IAEA-336	Lichen	0.20± 0.04	105
Pb	CRM008–050	Soil	95.3± 5.3	101.4
	IAEA-336	Lichen	4.9± 0.6	93.9
Cd	CRM008–050	Soil	0.82±0	103.6
	IAEA-336	Lichen	0.117± 0.017	98.2
